# Host Defense against Tumor

**DOI:** 10.31662/jmaj.2020-0048

**Published:** 2020-07-13

**Authors:** Masaki Mori

**Affiliations:** 1 Department of Surgery, Kyushu University, Fukuoka, Japan

**Keywords:** colon cancer, histology, invasion, metastasis, prognosis

In Japan, death from colorectal cancer has become the second leading cause among cancer deaths, and there were 50,628 deaths from colorectal cancer in 2018. Liver metastasis is a necessary determinant of colorectal cancer patients, and many studies have clarified prognostic factors associated with survival after resection of liver metastasis. They can be grouped into three categories, namely, tumor factors (TNM or Dukes stage of primary tumor, number of lymph node metastases, size and number of liver metastases, and preoperative CEA level), patient factors (age, sex, CRP level, and neutrophil/lymphocyte ratio), and treatment factors (surgical procedure, resection margin, and blood transfusion).

In this issue, Ueno et al. added another information to prognostic factors of colorectal liver metastasis and showed that survival after resection of liver metastasis from colorectal cancer is not associated with T or N stage but with primary tumor histology, including poorly differentiated clusters and desmoplastic reaction ^(1)^. They concluded that histologic features expressed in the primary tumor predict oncological outcome after resection of liver metastasis and are useful for identifying high-risk patients with early relapse after hepatic resection.

Interaction between tumor and host is a necessary aspect of host defense against tumor and must be emphasized from a viewpoint of surgical pathology. In 1960, Imai of Kyushu University paid attention to advancing margin of the tumor and proposed a novel prognostic classification abbreviated as CPL: C refers to cirrhotic sprouting (invading mode expressed by the extent of sprouting tumor cells); P refers to progressive sprouting (invasion without reactive fibrous tissue in their surroundings); and L refers to lymphatic and blood vessel permeation ^(2)^. In his study, the CPL classification was associated with the prognosis of patients who underwent resection of gastric and uterine cancers.

Another stromal aspect of host defense mechanism against tumor is exudative reaction, such as lymphocytic infiltration or lymphoid stroma ^(3), (4)^. In 1976, Watanabe et al. demonstrated that the presence of nondesmoplatic stroma infiltrated uniformly with abundant lymphocytes and plasma cells throughout the entire area of tumor showed high survival rate among 1,041 patients undergoing gastrectomy for cancer. In 1986, Jass (St Mark’s Hospital in London) evaluated 447 specimens of rectal cancer and showed that 5 year survival rates for pronounced, moderate, and little lymphocytic infiltration were respectively 92%, 65%, and 36%. His study first clarified that lymphocytic infiltration affected survival independent from other pathologic variables.

In the era of molecular biology or genetic pathology, researchers were prone to investigate the tumor pathology by the novel and evolving technology rather than the classical histologic examination.

Our great seniors reminded us that tumor tissue observation and evaluation simply via optical microscope is necessary in cancer study. In this sense, traditional histologic study based on hematoxylin-and-eosin stained slides by Ueno et al. is essential and suggestive for young readers of this journal, including researchers, pathologists, surgeons, and clinicians. Open your eyes, and look up to the skies and see (excerpt from Bohemian Rhapsody by Freddie Mercury) ^(5)^.

## Article Information

### Conflicts of Interest

None

### Disclaimer

Masaki Mori is one of the Associate Editors of JMA Journal and on the journal’s Editorial Staff. He was not involved in the editorial evaluation or decision to accept this article for publication at all.

## References

[1] Ueno H, Konishi T, Ishikawa Y, et al. Primary tumor histology affects oncological outcomes independently of the anatomic extent of disease in colorectal liver metastasis. JMA Journal. 2020;3(3):240-50. 10.31662/jmaj.2018-0004 PMC7590386 33150258

[2] Imai T. Growth patterns in human carcinoma: their classification and relation to prognosis. Obstet Gynecol. 1960;16(3):296-308. 13717558

[3] Watanabe H, Enjoji M, Imai T. Gastric carcinoma with lymphoid stroma: its morphologic characteristics and prognostic correlations. Cancer. 1976;38(1):232-43. 947518 10.1002/1097-0142(197607)38:1<232::aid-cncr2820380135>3.0.co;2-4

[4] Jass JR. Lymphocytic infiltration and survival in rectal cancer. J Clin Pathol. 1986;39(6):585-9. 3722412 10.1136/jcp.39.6.585 PMC499954

[5] Freddie Mercury, Queen. "Bohemian Rhapsody", A Night at the Opera. EMI. 1975.

